# Tubercular Peritonitis with Ascites Following Measles—Treatment

**Published:** 1890-12

**Authors:** L. H. Luce

**Affiliations:** West Tisbury, Mass.


For Daniel's Texas Medical Journal.

### A CASE OF TUBERCULAR PERITONITIS, WITH ASCITES FOLLOWING MEASLES—TREATMENT.

BY L. H. LUCE, M. D., WEST TISBURY, MASS.

THE following case is interesting from the severity of the disease following a mild attack of rubeola, and from the perfect recovery by medical treatment alone.

At the present time the treatment of diseases of the peritoneum is chiefly considered from the surgical standpoint, and laparotomy has a large number of followers who seem to completely ignore medical treatment. Two cases, recently published in the *Boston Medical Journal*, under the heading "Two Cases of Laparotomy for Tubercular Peritonitis," may be considered a fair presentation of the subject from a surgical standpoint, and as representing the present views of the profession. Although one case gives no chance for statistical deductions, it may well lead one to consider whether the present craze for operative interference in so sensitive a tissue as the peritoneum has not swung altogether too strongly in the opposite direction.

The patient, a female, of lymphatic temperament, aged 22, was attacked with rubeola the 25th of March, 1888. The family history was bad. Her mother having died of valvular disease of the heart, following chronic rheumatic arthritis, and which was complicated with interstitial pneumonia, involving the greater portion of both lungs. Her father died of phthisis pulmonalis, following chronic pleuritis, with extensive effusion into left pleural cavity. An aunt on the paternal side also died of chronic pleuritis. Her grandfather on the maternal side died suddenly of angina pectonis. One of her uncles on the same side died of phthisis pulmonalis, one of valvular disease of heart with pericarditis, and another of empyemia. The patient herself, about a year before her attack of measles, had chronic pleuritis, for which she was tapped, and over two quarts of fluid withdrawn. From this she made a perfect recovery. March 3, patient was married, and about three weeks after came with a mild attack of rubeola, the eruption appearing at the usual time, but not making its appearauce as abundantly as usual. Patient was about all the time and attending to her household duties, but contrary to advice. Four weeks after recovery from the measles she appeared at my office, complaining of the enlargement of the abdomen and "bloating." She had lost greatly in flesh and was pale and anæmic. Pulse 120, respiration 30 per minute.

An examination of the abdomen revealed an enlargement to the ensiforme cartilage, equally distended, very full and tense. Points of excessive tenderness at different parts of abdomen, but principally on the left side, near umbilicus, and in the left iliac region. Small nodular enlargements to upper and outer side of umbilicus were plainly detected. A distinct wave of fluid clearly appreciable even to the patient herself. Appetite was gone, bowels constipated. Stethoscopic examination of heart and lungs gave negative results. A specimen of urine, brought next day, also gave negative results on careful examination. Hepatic dullness normal. No jaundice or evidence of hepatic complications. The possible question of pregnancy was also to be considered. An examination revealed a perfectly normal uterus, os of normal hardness, and a probe, carefully passed, gave the dimension of the uterine cavity as about three inches. Menstruation was regular, and entire absence of morning sickness.

The diagnosis of this case must be based upon a close examination of the history of the case, the predisposing and exciting causes, and the assembly of local symptoms. The exceedingly bad history of the patient; her family a markedly tuberculous one. Her having had chronic pleuritis herself before the present attack. The extreme tenderness of the abdomen, with marked glandular enlargement, the ascites, with entire absence of organic disease of heart, liver and kidneys.

Without entering into a detailed account of the history, treatment, etc., we will simply state it in a general way. The constitutional treatment consisted, at first, of fairly large doses oi potassii iodide, 20 gr. doses four times a day, after meals and at bed time. This was continued for about tour weeks, reducing and increasing the dose according to its constitutional effects. At the same time small fly blisters were repeatedly applied to the abdomen over the points of tenderness. Finally teaspoonful doses of syr. ferri iodide were substituted for the potassii iodide, and given four times daily. The tr. of iodine was also substistuted for the fly blisters. Mild aperients of pil rhei. co. were given from time to time and diuretics of fl. ext. of buchu and potassii acetate were employed during the continuance of the ascites. As soon as the pulse was diminished in force and frequency, and the tenderness had subsided, a generous diet of eggs, milk and animal broths was allowed.

The patient made a perfect recovery and is to-day attending to her household duties apparently as well as ever.

Cases of tubercular peritonitis are at the present time subjected to laparotomy, and this case had all the indications usually enumerated as indicating this operation. In the article referred to at the commencement of this article, the following are enumerated as some of the indications for operation:

First—Indicating operation: 1. Abundant *ascites*. 2. Absence or small amount of *solid masses*. 3. Disease *primary* in the *peritoneum*. 4. Encysted disease. 5. Intestinal obstruction.

*Contra indicating operation*—1. Absence of ascites. 2. Abundant masses or strands. 3. Secondary to advanced tuberculosis. 4. Intestinal ulcers.

The opinion of authorities in regard to the question of operative interference is quite decisive. Taylor and Gail state that therapeutic changes are induced in the peritoneum by simply opening the cavity aud cleansing and washing out the cavity and inserting a drainage tube. Osler relates cases, where, after death from phthisis, the traces of tubercle in the peritoneum, cured, or in the process of cure were formed. Spaeth, Welch and Rochowneck hold contrary opinions.

It is far from proved that laparotomy gives us anythiug to hope for in tuberculous disease of the peritoneum, and in a disease where constitutional conditions, back of the local manifestations, are at work, it would seem unphilosophical to resort to so formidable an operation.

